# Valuing Happiness is Not a Good Way of Pursuing Happiness, but Prioritizing Positivity is: A Replication Study

**DOI:** 10.5334/pb.1036

**Published:** 2021-11-10

**Authors:** Michel Hansenne

**Affiliations:** 1 University of Liège, Department of Psychology, Psychology & Neuroscience of Cognition Research Unit, BE

**Keywords:** Prioritizing positivity, Valuing happiness, Well-being, Depression

## Abstract

Numerous studies confirm the merits of positive psychology. However, an emerging literature brings nuances, with one particular question concerning the impact of pursuing happiness: is this always positive? Some data suggest that an excessive level of valuing happiness may partly diminish the happiness felt, but that prioritizing positivity may increase subjective well-being. The aim of the present study was to replicate these findings. Participants (N = 683, 75% female) completed the prioritizing positivity scale (PPS) and the valuing happiness scale (VHS), as well as four well-being scales: the subjective happiness scale (SHS), the satisfaction with life scale (SWLS), the psychological well-being scales (PWB) and the center for epidemiological studies – depression (CES-D). Regression analyses showed that prioritizing positivity was positively associated with subjective happiness, life satisfaction and psychological well-being, but that it was negatively linked to depression. Opposite results partly emerged for valuing happiness. This cross-sectional study confirms that the way people pursue happiness, by prioritizing positivity or valuing it, may promote or partly hinder well-being.

## Introduction

Everybody wants to be happy, and several lines of evidence clearly demonstrate the positive benefits of happiness on various important outcomes, such as subjective happiness, life satisfaction, psychological well-being, and physical health (Diener et al., 1999; Fredrickson, 1998; Lyubomirsky et al., 2005). In addition, since the development of positive psychology, research has stressed that simple positive activities might increase well-being (Layous et al., 2014; Lyubomirsky & Layous, 2013; Mongrain & Anselmo-Matthews, 2012; Sin & Lyubomirsky, 2009). However, an emerging literature brings nuances, and one question in particular concerns the impact of pursuing happiness: is this always positive? Pursuing happiness may be counterproductive for several reasons: because people might have high unrealistic standards that are too difficult to attain, because they might focus continuously on their level of happiness instead of just behaving normally, or because they might use inefficient methods to achieve happiness (Ford & Mauss, 2014; Gruber et al., 2011). In the same vein, research suggests that positive activities may undermine happiness, depending on how, when, and why these activities are carried out (Fritz & Lyubomirsky, 2018; Sheldon & Lyubomirsky, 2012). Being happy is a delicate art, requiring the right tools to achieve it, and there are wrong ways to attain happiness.

### The paradoxical effect of valuing happiness

Recent evidence has suggested that valuing happiness, i.e. the tendency to want to be happier most of the time in most circumstances, may lead to a decrease in positive feelings and happiness, as well as to an increase in depressive symptoms and loneliness (Ford et al., 2014; Gentzler et al., 2019; Mauss et al., 2011; Mauss et al., 2012). Mauss and colleagues (2011) developed a short scale to assess the valuing of happiness (valuing happiness scale, VHS) and used this to demonstrate that valuing happiness was related to a lower hedonic balance (i.e., relative experience of positive and negative affect), lower psychological well-being and lower life satisfaction, but higher levels of depressive symptoms. Moreover, when valuing happiness was experimentally induced just before the participants watched a positive film clip, the feelings of happiness experienced were reduced, suggesting a causal relationship between valuing happiness and experiencing it. A subsequent study has described a significant relationship between valuing happiness and reported levels of loneliness, particularly when experiencing stressful events in daily life (Mauss et al., 2012). Higher levels of self-reported loneliness were also reported in the same study after an experimental manipulation of valuing happiness. In another study, remitted depressive patients who valued happiness exhibited a greater severity of depressive symptoms, based on both self-reported and clinician-rated depressive symptoms (Ford et al., 2014). Gentzler et al. (2019) found a positive relationship between depressive symptoms and valuing happiness in young people (7 to 18 years). However, negative associations between valuing happiness and well-being measures were mixed. While valuing happiness was negatively associated with life satisfaction in adolescents aged 14 to 18 years, there was no relationship with subjective happiness among these adolescents. In a wider age range (12 to 18 years), the authors reported that valuing happiness was unrelated to well-being measures, but that age moderated the link – valuing happiness was related to greater purpose in life for younger adolescents (12 to 13.8 years), but to lower purpose in life for older adolescents (16.8 to 18 years), and valuing happiness was unrelated to life satisfaction for younger adolescents (12 to 13.2 years), but to lower satisfaction for older adolescents (15.9 to 18 years). Taken together, these findings provide evidence suggesting a paradoxical effect of valuing happiness: trying to be happy might backfire (Ford & Mauss, 2014).

It would be interesting to consider whether the detrimental motivation to want to be happy on well-being found in individualistic societies could be favourable in collectivistic societies, i.e., are there cultural differences in the link between valuing happiness and well-being? This was tested in four regions of the world that differ significantly on this point, namely the United States (Denver), Germany (Tübingen), Russia (Moscow and Tomsk), and East Asia (Kyoto and Taipei) (Ford et al., 2015). Results indicated that valuing happiness was negatively correlated with a composite index of well-being in the American sample (r = –.26) but not in Germany (r = –.11, NS). The associations were, in contrast, positive in Russia (r = .31) and East Asia (r = .25). Moreover, both collectivistic countries envisaged achieving happiness through social engagement, which was not the case in the two individualistic countries.

### Prioritizing positivity as a successful way to happiness

Wanting to be happy as an absolute goal may can lead people to be less happy, valuing happiness can backfire, and there is no single way to attain happiness. It is very important that the activities people choose to do, in order to be happy, are reasonable, not dictated by excessive or unattainable standards. These activities should also be tailored (e.g., person-activity fit) for the person seeking happiness (Layous & Lyubomirsky, 2014). One way to increase happiness is to select situations that can induce more positive emotions, and consequently increase happiness. Situation selection is one of the five emotion regulation processes proposed by Gross (1998), in which individuals can select environments that allow or prevent the occurrence of specific emotions.

In line with the effectiveness of situation selection as an emotion regulation strategy (Livingstone & Isaacowitz, 2015; Van Bockstaele et al., 2020), Catalino et al. (2014) have suggested that, if people prioritize positivity when making decisions about their daily life activities, this could increase happiness. They postulated that selecting environments and activities that give rise to positive feelings would be a successful means to achieve happiness. To test this hypothesis, a short self-report instrument was developed to assess individual differences on prioritizing positivity, the prioritizing positivity scale (PPS) (Catalino et al., 2014). Because prioritizing positivity and valuing happiness are conceptually linked, the authors predicted that PPS and VHS would have the opposite effects on happiness. Results showed that PPS was positively associated with positive emotions and life satisfaction, but negatively associated with negative emotions and depressive symptoms. In contrast, VHS exhibited the opposite associations: negative relationships with positive emotions and life satisfaction, but positive associations with negative emotions and depressive symptoms. These results confirm the deleterious effect of valuing happiness (Ford et al., 2014; Mauss et al., 2011). In addition, when both PPS and VHS were introduced simultaneously as predictors, the effects were stronger (Catalino et al., 2014). This suggests that prioritizing positivity could induce a negative impact on happiness, as captured by the shared variance with VHS. And when this negative part is removed, this shows how efficient it is to make positivity a priority when choosing the activities we want to do. Conversely, valuing happiness contains a positive part, as captured by the shared variance with PPS, and without this positive part, valuing happiness is shown to be particularly deleterious.

The positive association between giving priority to the positive and self-reported experience of well-being has been replicated in a longitudinal study carried out on secondary school students (Datu & King, 2016). A life-span study conducted on people ranging from 17 to 87 years has also confirmed that prioritizing positivity increases positive emotions and decreases negative ones (Littman-Ovadia & Russo-Netzer, 2018). More specifically, age was found to moderate the relationship between PPS and emotion. Older people who prioritized positivity experienced more positive emotions than those who did not. On the other hand, among young people who gave priority to the positive, this attitude did not influence their experience of positive emotions. Regarding negative emotions, the more young people gave priority to the positive, the less negative emotions they experienced, while the relationship was weaker among older people.

### The present study

The main purpose of the present study was to replicate the positive relationship of prioritizing positivity and the negative association of valuing happiness on well-being measures, as reported in previous studies (Catalino et al., 2014; Datu & King, 2016; Ford et al., 2014; Littman-Ovadia & Russo-Netzer, 2018; Mauss et al., 2012). Based on previous findings, it was hypothesized that prioritizing positivity (PPS) would positively predict well-being measures and negatively predict depressive symptoms, while the opposite results were expected concerning valuing happiness (VHS). The two scales were also expected to have a shared common variance, which, when controlled for, would lead to an increase in the specific effects of each scale, as reported by Catalino et al. (2014). Moreover, in order to obtain a more comprehensive estimation of well-being, a measure of psychological well-being (eudaimonic well-being) was also included, in addition to measures of life satisfaction and subjective happiness (hedonic well-being) (Ryan & Deci, 2001). The hedonic approach to well-being refers to a pleasant life with the maximization of happiness and the minimization of pain, and the eudaimonic aspect of well-being corresponds to a meaningful life and self-actualization. In addition, given that previous studies have been conducted mainly in the United States, where the pursuit of happiness is highly valued, although there is no evidence that the pursuit of happiness is stronger in the United States than in Belgium, the present study will further explore the relationship between the pursuit of happiness and well-being in a large sample of this Western European country.

## Methods

### Participants

The sample consisted of 683 participants comprising 394 students (75% female), with a mean age of 28.5 years (ranging from 18 to 72 years, SD = 12.94) from the French-speaking part of Belgium. Undergraduate students were invited to participate in the study as part of a course. Staff from the academic community were invited by e-mail to be part of the study. Individuals were also recruited through social networks. No compensation was given to the participants for being part of the study. The participants completed online both the VHS and the PPS scales, as well as four well-being scales. The ethics committee of the Psychology Faculty approved the protocol, and the participants gave their informed consent to participate in the study.

### Measures

#### Valuing Happiness Scale

The valuing happiness scale (VHS) was developed by Mauss et al. (2011) to assess the tendency to value happiness extremely and most of the time. The scale is composed of seven items, such as “How happy I am at any given moment says a lot about how worthwhile my life is”, “If I don’t feel happy, maybe there is something wrong with me”, “Feeling happy is extremely important to me”, or “I am concerned about my happiness even when I feel happy”. Participants had to respond on a 7-point scale ranging from strongly disagree (1) to strongly agree (7). The scale was translated into French by two independent scholars and adapted after checking by a native speaker (a = .65 in the present study).

#### Prioritizing Positivity Scale

The prioritizing positivity scale (PPS) was developed by Catalino et al. (2014) to assess the extent to which people view experiencing positive emotions to be a major factor they consider when planning for activities in their daily life. People who prioritize positivity choose activities in their daily life that they anticipate will lead to positive feelings. The scale comprises six items, such as “I structure my day to maximize my happiness”, “A priority for me is experiencing happiness in everyday life”, or “What I decide to do with my time outside of work is influenced by how much I might experience positive emotions”. Participants had to respond on a 9-point scale ranging from strongly disagree (1) to strongly agree (9). The scale was translated into French by two independent scholars and adapted after checking by a native speaker (a = .75 in the present study).

#### Well-being scales

*Subjective Happiness Scale*. Overall happiness was assessed using the French version of the subjective happiness scale (SHS; Lyubomirsky & Lepper, 1999; Kotsou & Leys (2017) for the French version). This instrument, composed of four 7-point items (e.g., “In general, I consider myself: not a very happy person (1) – a very happy person (7)”), provides a global subjective assessment of whether an individual is a happy or an unhappy person (a = .84 in the present study).

*Satisfaction With Life Scale*. The satisfaction with life scale (SWLS; Diener et al., 1985) is an instrument evaluating the extent to which people judge their lives to be satisfactory or not. Participants completed the French version of the scale (Blais et al., 1989; a = .86 in the present study) by giving their agreement or disagreement regarding five items on a 7-point scale, ranging from strongly disagree (1) to strongly agree (7), such as “The conditions of my life are excellent”, or “So far I have gotten the important things I want in life”.

*Psychological Well-Being Scales.* Psychological well-being was assessed using the French version of the 18-item psychological well-being scales (PWB; Ryff & Keyes, 1995; Bouffard & Lapierre (1997) for the French version; a = .75 in the present study). Participants were asked to specify the degree to which they agreed or disagreed with each statement on a 6-point scale, ranging from strongly disagree (1) to strongly agree (6), such as “I have confidence in my opinions, even if they are contrary to the general consensus”, “I have a sense of direction and purpose in life”, or “I am quite good at managing the many responsibilities of my daily life”. The instrument is composed of 6 scales (autonomy, environmental mastery, personal growth, positive relations, purpose in life, and self-acceptance), and a total score is also provided. In lines with previous studies (Garcia, 2011; Tamir & Ford, 2012), only the total score was considered.

*Center for Epidemiological Studies–Depression.* The Center for Epidemiological Studies–Depression (CES-D; Radloff, 1977) is a 20-item self-reported measure assessing depressive symptoms. Participants completed the French version of the scale (Fuhrer & Rouillon, 1989, a = .74 in the present study). They were asked to indicate how often they had experienced the symptoms described in each item in the past week on a 4-point scale (0 = rarely or none of the time (less than 1 day), 1 = some or a little of the time (1–2 days), occasionally or a moderate amount of time (3–4 days), 4 = most or all of the time (5–7 days)).

## Results

Correlation analyses showed no significant association between either PPS (r = .04, *p* = .33) or VHS (r = .06, *p* = .15) and age. In addition, age was not significantly associated with PWB (r = .05, *p* = .19) or SWLS (r = .06, *p* = .15), and there was a weak association with SHS (r = .17, *p* < .001) and CES-D (r = –.13, *p* < .001). So, age was not introduced as a variable in further analyses. Gender analyses revealed only modest gender differences for PPS (t = 3.78, *p* <.001, *d* = .34) and CES-D (t = 2.70, *p* = .01, *d* = .24), such that women scored higher on prioritizing positivity and depressive symptoms. However, when gender was included as a covariate, the results were not significantly different. Thus, this variable was not considered in the presentation of the results.

A total of 12 regressions were conducted (***Table 1***). In order to examine whether prioritizing positivity related to well-being for the participants, regression analyses were conducted with PPS as the predictor of the four well-being measures: satisfaction with life, subjective happiness, psychological well-being, and depressive symptoms (***Table 1***, column 1). Results showed positive associations between PPS and the first three measures: satisfaction with life, subjective happiness, and psychological well-being. Analyses also revealed a negative association between prioritizing positivity and depressive symptoms, meaning that the more people made decisions in their daily life based on the anticipation of experiencing positive feelings, the less they exhibited depressive symptoms.

**Table 1:** Regression analyses (standardized coefficients) of well-being measures on valuing happiness and prioritizing positivity (in columns 3 and 4, valuing happiness and prioritizing positivity are simultaneously entered as predictors).


WELL-BEING MEASURES	PRIORITIZING POSITIVITY	VALUING HAPPINESS	DIRECT EFFECT OF PRIORITIZING POSITIVITY (CONTROLLING FOR VALUING HAPPINESS)	DIRECT EFFECT OF VALUING HAPPINESS (CONTROLLING FOR PRIORITIZING POSITIVITY)

Satisfaction with life	.27***	–.15***	.39***	–.30***

Subjective happiness	.35***	–.14***	.48***	–.32***

Psychological well-being	.27***	–.21***	.41***	–.37***

Depressive symptoms	–.17***	.27***	–.32***	.39***


*Note*: *** p < .001.

The same regression analyses were conducted with valuing happiness as the predictor (VHS) (***Table 1***, column 2). Results revealed a positive association between valuing happiness and depressive symptoms, as assessed by the CES-D. In addition, the other three well-being measures – satisfaction with life, subjective happiness, and psychological well-being – were found to be negatively predicted by valuing happiness (VHS).

Since prioritizing positivity (PPS) was found to be positively correlated with valuing happiness (VHS) (r = .39, *p* < .001), further regression analyses were conducted for each well-being variables on PPS and VHS measures introduced as concurrent predictors (columns 3 and 4). Those analyses allowed to consider two effects: firstly, the direct effect of PPS on well-being measures, after controlling for the effect of valuing happiness and secondly, the direct effect of VHS on well-being measures, after controlling for the effect of prioritizing positivity. The results revealed significant positive associations between PPS and the first three measures: satisfaction with life, subjective happiness, and psychological well-being. They also showed a negative association between PPS and the fourth measure, depressive symptoms. In each case, higher associations were obtained for the concurrent PPS and VHS predictors than when PPS introduced as a unique predictor (***Table 1***, column 3). Conversely, concerning valuing happiness, results showed direct negative effects of VHS on satisfaction with life, subjective happiness, and psychological well-being, but a positive effect on depressive symptoms (***Table 1***, column 4).

To investigate which well-being variables best predicted VHS and PPS, the four well-being variables were entered simultaneously into a regression model. Regression analyses showed that CES-D had the stronger significant effect on VHS (b = .27, *p* < .001), and that SHS and CES-D had the most significant effects on PPS (respectively: b = .34, *p* < .001; b = –.21, *p* < .001).

## Discussion

The results of this study confirm that prioritizing positivity is associated with well-being, showing moderate positive associations with subjective happiness, life satisfaction and psychological well-being, as well as a slight negative association with depressive symptoms. These findings are in line with previous studies demonstrating the positive relationship on well-being outcomes of prioritizing the positive (Catalino et al., 2014; Datu & King, 2016; Littman-Ovadia & Russo-Netzer, 2018). Interestingly, the present results also extend previous findings by demonstrating the positive association on psychological well-being of prioritizing positivity (i.e., the eudaimonic component of well-being). A positive association between prioritizing positivity and one aspect of psychological well-being (i.e., positive relations) has been reported previously (Catalino et al., 2014), but this study included only this measure from the six psychological well-being scales of Ryff & Keyes (1995). Happiness is more than a pleasurable life, having a meaningful life and being self-actualized are also important (Ryan & Deci, 2001). Although there is a difference between the hedonic and eudaimonic parts of well-being, the results of the present study revealing the same associations between VHS and PPS and the measures of subjective and psychological well-being are interesting. Valuing happiness is not only related to a reduction in pleasure, but may hinder self-realization, the opposite pattern being observed for prioritizing positivity. In addition, the results confirm that PPS may include a negative side, as captured by the shared variance with VHS. Moreover, in line with the results of Catalino et al. (2014), it was found that when this negative part was removed, the results demonstrated even further the efficiency of making positivity a priority when deciding what activities we want to do.

One may question, however, the association found between happiness and prioritizing positivity. Is this possible for everyone? Does prioritizing positivity really increase happiness, or does happiness allow us to give priority to the positive? Perhaps prioritizing positivity is only possible when people have the freedom and the ability to decide to give priority to activities that give rise to positive feelings. One can speculate that these people are probably already happy, and that giving priority to positivity makes them even happier. Conversely, making positivity a priority is not possible for everybody, particularly for people who have difficulties identifying positive situations in their lives, who have poor emotional competences, or who cannot select sufficient positive situations in order to prevent hedonic adaptation. Indeed, as has been suggested by the Adaptation Hedonic Prevention model, an effective way to reduce hedonic adaptation to the positive situations we experience is to diversify the variety of positive activities we do (Sheldon & Lyubomirsky, 2012; Sheldon et al., 2013). But do all people have this freedom? It could be interesting in further studies to include variables assessing how much people have the opportunity to choose positive situations (e.g., education, income) and whether that moderates the relationship of PPS to well-being.

On the other hand, the present findings confirm the positive association between valuing happiness and depressive symptoms; that is, wanting to be happy as an absolute goal can induce negative feelings (Catalino et al., 2014; Ford et al., 2014; Mauss et al., 2012). In addition, the results indicate slight associations between valuing happiness and well-being. This confirm previous findings showing a negative association between valuing happiness and well-being measures, such as life satisfaction or subjective happiness (Catalino et al., 2014; Mauss et al., 2011). Previous studies have however reported mixed results supporting the hypothesis of the negative association between valuing happiness and well-being (Gentzler et al., 2019), or have demonstrated that only some items from VHS are negatively associated with well-being (Luhmann et al., 2015). When VHS was introduced with PPS as a simultaneous predictor, the present findings demonstrate stronger negative impact of valuing happiness on well-being. This confirms that VHS contains a positive part, as captured by the shared variance with PPS, and that without this positive part, valuing happiness is particularly deleterious (Catalino et al., 2014).

Therefore, the current results confirm and extend what has been shown in previous studies, namely that valuing happiness leads to feelings of depression and loneliness, and partly diminishes the happiness felt. This relationship does not appear to be specific to the North American population. If the standards are too high, the level of satisfaction will be impacted, and spending a considerable amount of time in the quest for happiness can distract us from our social relationships. Always wanting to be happier can frustrate people because the level of satisfaction is perpetually pushed back, leading to a feeling of frustration, mainly in positive situations (Gruber et al., 2011; Mauss et al., 2011). Negative situations represent a different case, since it is normal for these negative situations not to produce happiness. So, we accept in these situations that we may not be happy, since it is the situation that induces this state and not ourselves. It is therefore important to point out that valuing happiness does not mean that we will be happy. On the other hand, valuing happiness is not always counterproductive. As suggested by Mauss et al. (2011), valuing happiness can have a positive outcome when people have the skills and the right tools to achieve a high level of happiness – essentially, when they demonstrate good emotion regulation strategies and a good level of global functioning. Indeed, some studies have reported that better emotion regulation strategies are linked to well-being (Blanchard-Fields & Coats, 2008; Quoidbach et al., 2010), and Quoidbach et al. (2015) have suggested that emotion regulation strategies could be view as a conceptual framework explaining the effects of positive interventions on well-being. As suggested by Catalino et al. (2014), valuing happiness is related to the attempt to directly regulate positive or negative emotions, while prioritizing positivity refers more to trying to experience situations that elicit positive emotions. Therefore, one can argue that the opposite influences of valuing happiness and prioritizing positivity on well-being may suggest that trying to influence the valence of an emotional experience may be more difficult that altering the occurrence of situation that elicit a positive emotional reaction.

Several studies have suggested that experiencing negative and positive emotions is beneficial for well-being (Dejonckheere et al., 2019; Quoidbach et al., 2014). Negative emotions are not always bad (Houle & Philippe, 2020; Tamir, 2016). Experiencing both positive and negative emotions also means that individuals are attentive to their emotions and have emotional clarity to facilitate their thoughts. It could be argued that valuing happiness could be associated with a desperate search for happiness and the avoidance of any negative emotions, such as sadness or shame. In the world of happy people, negative emotions are not wanted. This may be the reason why valuing happiness is negatively associated with well-being. However, prioritizing positivity could be a strategy in which the negative side of a situation is recognized, which could result in greater overall well-being. For example, age was found to significantly moderate the relationships of prioritizing positivity with positive and negative emotions (Littman-Ovadia & Russo-Netzer, 2018), meaning that prioritizing positivity reduced negative emotions in young people, but increased positive emotions in older people.

The present findings are consistent with those observed in the United States, but differ from the results observed in Germany, where the association between valuing happiness and well-being, albeit negative, was not significant (Ford et al., 2015). Since culture has been found to modulate the relationship between valuing happiness and well-being, one explanation given by the authors is that there is evidence suggesting that Germany may be somewhat more collectivistic than the United States (Koopmann-Holm & Matsumoto, 2011), so that valuing happiness does not have a negative effect on well-being. This explanation can probably be given to justify the difference between Belgium and Germany, since the former is more individualistic than the latter (Perlitz & Seger, 2004).

It should be noted that no a priori power analysis was performed and that the present study is most likely overpowered, leading to very significant results. Comparisons with size effects of the original study of Catalino et al. (2014) are therefore of interest. All effect sizes (i.e., standardized regression coefficients) reported in the present study are smaller than those found in the study of Catalino et al. (2014), but they can be considered as moderate and the differences are not large (respectively –.30 and –.34 for the direct negative effect of VHS on satisfaction; .39 and .40 for the direct positive effect of VHS on depressive symptoms; and respectively .39 and .45 for the direct positive effect of PPS on satisfaction; –.32 and –.37 for the direct negative effect of PPS on depressive symptoms).

In conclusion, the present study confirms on a larger sample that the way in which people seek happiness, by prioritizing positivity or valuing happiness, is related to well-being. It is important to underline, however, that the sample in the present study was an online sample comprising 58% of students and consisting of mostly females (75%). Further studies would therefore need to be carried out on a more representative sample. In addition, although the present results are consistent with the hypotheses that valuing happiness can lead to less happiness, and that, on the contrary, prioritizing positivity can induce more happiness, the study is correlational and therefore open to other interpretations. Indeed, it could be that unhappy people may simply find it too difficult to engage in activities that lead to pleasurable emotional experience, or they could also value happiness to a greater extent.
